# Human BCDIN3D Is a Cytoplasmic tRNA^His^-Specific 5′-Monophosphate Methyltransferase

**DOI:** 10.3389/fgene.2018.00305

**Published:** 2018-08-03

**Authors:** Kozo Tomita, Yining Liu

**Affiliations:** Department of Computational Biology and Medical Sciences, Graduate School of Frontier Sciences, The University of Tokyo, Kashiwa, Japan

**Keywords:** Bicoid interacting 3 domain containing RNA methyltransferase, methylation, tRNA, breast cancer, protein synthesis

## Abstract

Bicoid interacting 3 domain containing RNA methyltransferase (BCDIN3D) is a member of the Bin3 methyltransferase family and is evolutionary conserved from worm to human. BCDIN3D is overexpressed in breast cancer, which is associated with poor prognosis of breast cancers. However, the biological functions and properties of BCDIN3D have been enigmatic. Recent studies have revealed that human BCDIN3D monomethylates 5′-monophsosphate of cytoplasmic tRNA^His^ *in vivo* and *in vitro*. BCDIN3D recognizes the unique and exceptional structural features of cytoplasmic tRNA^His^ and discriminates tRNA^His^ from other cytoplasmic tRNA species. Thus, BCDIN3D is a tRNA^His^-specific 5′-monophosphate methyltransferase. Methylation of the 5′-phosphate group of tRNA^His^ does not significantly affect tRNA^His^ aminoacylation by histidyl-tRNA synthetase *in vitro* nor the steady state level or stability of tRNA^His^ *in vivo*. Hence, methylation of the 5′-phosphate group of tRNA^His^ by BCDIN3D or tRNA^His^ itself may be involved in certain unknown biological processes, beyond protein synthesis. This review discusses recent reports on BCDIN3D and the possible association between 5′-phosphate monomethylation of tRNA^His^ and the tumorigenic phenotype of breast cancer.

## Introduction

Bicoid interacting 3 domain containing RNA methyltransferase (BCDIN3D) contains an S-(5′-adenosyl)-L-methionine (AdoMet) binding motif, and is homologous to a conserved family of eukaryotic protein methyltransferases acting on RNA-binding proteins (Zhu and Hanes, 2000). The BCDIN3D is evolutionary conserved and has been identified in various animals from worms to human (Xhemalce et al., 2012), however, its biological properties and functions are unclear. BCDIN3D mRNA overexpression has been reported in human breast cancer cells, which is associated with cellular invasion and poor prognosis in triple-negative breast cancer (Liu et al., 2007; Yao et al., 2016). The molecular basis of involvement of BCDIN3D in the tumorigenic phenotype of breast cancer has remained elusive. This review discusses recent studies on human BCDIN3D. We describe herein that a specific tRNA for histidine (tRNA^His^) is now identified as a primary target of BCDIN3D and discuss the association between the tumorigenic phenotype of breast cancer and the methylation of tRNA^His^ by BCDIN3D.

## How Does Human Bcdin3D Recognize Specific Rna?

Xhemalce et al. (2012) reported that BCDIN3D catalyzes dimethylation of 5′-monophosphate of specific precursor microRNAs (pre-miRNAs) (Xhemalce et al., 2012), such as tumor suppressor miR145 and miR23b (He et al., 2007; Shi et al., 2007; Sachdeva et al., 2009; Spizzo et al., 2010), using AdoMet as a methyl-group donor. Dimethylation of the 5′-monophosphate of pre-miRNA nullifies the negative charge at the 5′-terminal of pre-miRNA. Since Dicer recognizes the negative charge at the 5′-terminal of pre-miRNAs for efficient and accurate cleavage (Park et al., 2011), the dimethylation of 5′-phosphate of pre-miRNA inhibits subsequent processing. Consequently, mature miRNAs are down-regulated. They also reported that the depletion of BCDIN3D mRNA by specific shRNAs suppressed the tumorigenic phenotype of MDA-MB231 breast cancer cells (Xhemalce et al., 2012). Therefore, it was proposed that BCDIN3D promotes the cellular invasion of breast cancer cells by downregulating tumor suppressor miRNAs through dimethylation of the 5′-phosphate group of the corresponding pre-miRNAs. However, there are no apparent common features including primary or secondary structures among the corresponding pre-miRNAs of downregulated miRNAs in breast cancer cells. Thus, the mechanisms by which BCDIN3D recognizes only a specific group of pre-miRNAs and downregulates mature miRNAs in breast cancer cells are unclear.

## Cytoplasmic tRna^His^ is Co-Purified With Bcdin3D and Contains a 5′-Monomethylmonophosphate Group

To identify other potential RNA substrates of BCDIN3D *in vivo* and to elucidate the mechanism underlying the recognition and regulation of specific RNAs by BCDIN3D, recently, BCDIN3D-binding RNAs in human HEK293T cells were analyzed (Martinez et al., 2017). When BCDIN3D, expressed in HEK293T cells, was purified from the cell extracts, a distinct 70–80-nucleotie-long RNA molecule was co-purified with BCDIN3D protein.

**FIGURE 1:**
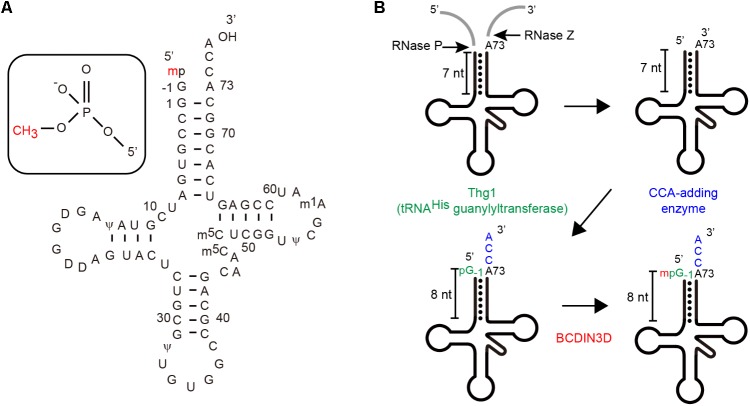
**(A)** The nucleotide sequence of human cytoplasmic tRNA^His^ including modified nucleosides. Human cytoplasmic tRNA^His^ in a clover-leaf structure, wherein the 5′-phosphate group is monomethylated (Rosa et al., 1983). **(B)** Maturation of human cytoplasmic tRNA^His^ (Gu et al., 2003; Betat et al., 2014) Cytoplasmic tRNA^His^ has an additional guanosine residue at position-1 (G_-1_) and an 8-nucleotide-long acceptor helix with G_-1_:A_72_ mis-pairing at the top of the acceptor helix (Rosa et al., 1983).

It was assumed that this co-purified RNA might be cytoplasmic tRNA^His^ (**Figure 1A**), since the nucleotide sequences of cytoplasmic tRNA^His^ from human and fruit fly reportedly contained a 5′-monomehtylphosphate group (Cooley et al., 1982; Rosa et al., 1983). Analysis of the RNA co-purified with BCDIN3D via RT-PCR and sequencing confirmed that cytoplasmic tRNA^His^ is co-purified with BCDIN3D from the cell extracts, but not other tRNAs, such as tRNA^Phe^. Subsequent direct analysis of the RNA via liquid chromatography and mass spectrometry (LC-MS) revealed that this RNA is cytoplasmic tRNA^His^. Moreover, the 5′-monophosphate of cytoplasmic tRNA^His^ was fully monomethylated, but not dimethylated at all. Furthermore, 5′-monophsophate of tRNA^His^ is reportedly fully monomethylated even under normal physiological conditions in HEK293T cells, as observed previously in cytoplasmic tRNA^His^ from HeLa cells (Rosa et al., 1983).

## Cytoplasmic tRna^His^ is Methylated by Bcdin3D *in Vitro*

The enzymatic activity of recombinant human BCDIN3D expressed in *E. coli* was examined using human cytoplasmic tRNA^His^ transcript as a substrate and S-(5′-adenosyl)-L- methionine (SAM) as a methyl-group donor *in vitro* (Martinez et al., 2017*).* Cytoplasmic tRNA^His^ transcript was reportedly efficiently methylated by BCDIN3D *in vitro*; however, unexpectedly, human pre-miR-145, which was previously reportedly dimethylated by BCDIN3D (Xhemalce et al., 2012), is hardly methylated under the same conditions assessed. The reaction products were further analyzed via LC-MS and it was confirmed that BCDIN3D monomethylates 5′-monophosphate of cytoplasmic tRNA^His^. Almost 100% of the tRNA^His^ reaction product comprised 5′-monomethylphosphate. Moreover, BCDIN3D does not dimethylate 5′-monophosphate of tRNA^His^ or pre-miR145 *in vitro*. Steady-state kinetics of methylation of these RNA substrates revealed that cytoplasmic tRNA^His^ is a greater than 2–3 orders of magnitude better substrate than pre-miR145.

BCDIN3D reportedly dimethylates pre-miR145 (Xhemalce et al., 2012). However, only a small fraction (less than 1%) of pre-miR145 substrates was methylated at the reaction end points (Xhemalce et al., 2012). The lower methylation of pre-miR145 by BCDIN3D is consistent with that in the recent study (Martinez et al., 2017). Furthermore, BCDIN3D reportedly transfers two methyl-groups from SAM to 5′-monophosphate of pre-miR145 (Xhemalce et al., 2012). This is inconsistent with the recent findings of Martinez et al. (2017), wherein neither tRNA^His^ nor pre-miR145 are dimethylated by BCDIN3D *in vitro*. Perhaps, the efficiency of dimethylation of 5′-phosphate of pre-miR145 by BCDIN3D would be much lower than that of monomethylation of 5′-phosphate of pre-miR145 *in vitro*. The previously observed methylation of pre-miR145 (Xhemalce et al., 2012) is at baseline levels, as compared with that in cytoplasmic tRNA^His^, and is not significant.

## Cytoplasmic tRna^His^ is Methylated by Bcdin3D *in Vivo*

BCDIN3D-knockout HEK293T cells, established via CRISPR/Cas9 editing, are viable, although they exhibit a slightly reduced growth rate than the parental cells (Martinez et al., 2017). Cytoplasmic tRNA^His^ isolated from BCDIN3D-knockout cells completely lost their methyl moiety at the 5′-monophosphate group, as evident from LC-MS. Exogenous expression of BCDIN3D in the BCDIN3D-knockout cell restored the 5′-monomethylphosphate modification of cytoplasmic tRNA^His^. Thus, the BCDIN3D is responsible for the monomethylation of 5′-monophosphate of cytoplasmic tRNA^His^ in HEK293T cells under normal physiological conditions. In BCDIN3D-knockout cells, no other RNAs except for cytoplasmic tRNA^His^, are significantly methylated by recombinant BCDIN3D *in vitro* (Martinez et al., 2017).

A recent study using HEK293T cells reported that BCDIN3D-knockout or BCDIN3D overexpression do not alter mature miR145 expression levels (Martinez et al., 2017), concurrent with the recent *in vitro* results showing that BCDIN3D does not dimethylate the 5′-monophosphate group of either tRNA^His^ or pre-miR145. Only pre-miRNA with 5′-dimethylated phosphate, but not pre-miRNA with 5′-monomethylated phosphate, is processed at lower levels by Dicer (Xhemalce et al., 2012). These observations also suggest that BCDIN3D does not dimethylate the 5′-phosphate group of pre-miR145.

Together with the recent results of *in vitro* methylation assays using recombinant BCDIN3D and tRNA^His^ transcript and the pre-miR145 transcript (Martinez et al., 2017), the primary target of BCDIN3D is cytoplasmic tRNA^His^ rather than pre-miRNAs. BCDIN3D displays monomethylation activity on the 5′-phosphate group of RNA. Considering the significantly lower activity of BCDIN3D toward pre-miR145 and that miR145 expression is not regulated by BCDIN3D *in vivo*, methylation of pre-miR145 probably does not occur in HEK293T cells. Under certain biological process or specific conditions in breast cancer cells, BCDIN3D might recognize specific pre-miRNAs, such as pre-miR145, through the regulatory factors which assist BCDIN3D in recognizing specific RNA species. Elucidation of the regulatory mechanism of specific pre-miRNA (di)methylation process by BCDIN3D in breast cancer cells awaits further study.

## tRna^His^ Recognition by Bcdin3D

Human cytoplasmic tRNA^His^ is matured through unique processes and has unique structural features among cytoplasmic tRNA species (Jackman et al., 2012; Betat et al., 2014; **Figure 1B**). After transcription by RNA polymerase-III, the 5′-leader and 3′-tailer sequences of precursor tRNA^His^ are cleaved. Thereafter, a single guanosine residue (G) is attached to the 5′-end (at position -1) in the 3′–5′ direction by tRNA^His^-specific guanylyltransferase (Thg1) (Gu et al., 2003; Jackman and Phizicky, 2006; Jackman et al., 2012) and the CCA is added at the 3′-end (positions 74–76) (Tomita and Yamashita, 2014). Consequently, the mature form of cytoplasmic tRNA^His^ has an 8-nucleotide-long acceptor helix with G_-1_:A_73_ mis-paring at the top the helix, while other cytoplasmic tRNAs have 7-nucleotide-long acceptor helices.

*In vitro* steady-state kinetics of methylation of mutant cytoplasmic tRNA^His^ transcripts by recombinant BCDIN3D revealed that BCIDN3D recognizes G_-1_, G_-1_:A_73_ mis-pairing at the top of the acceptor stem and 8-nucleotide-long extended acceptor helix. The minihelix of tRNA^His^ is also methylated efficiently by BCIN3D. Thus, BCDIN3D recognizes the unique structural features of cytoplasmic tRNA^His^, especially in the top-half region of tRNA^His^, and discriminates cytoplasmic tRHA^His^ from other tRNA species (Martinez et al., 2017).

**FIGURE 2:**
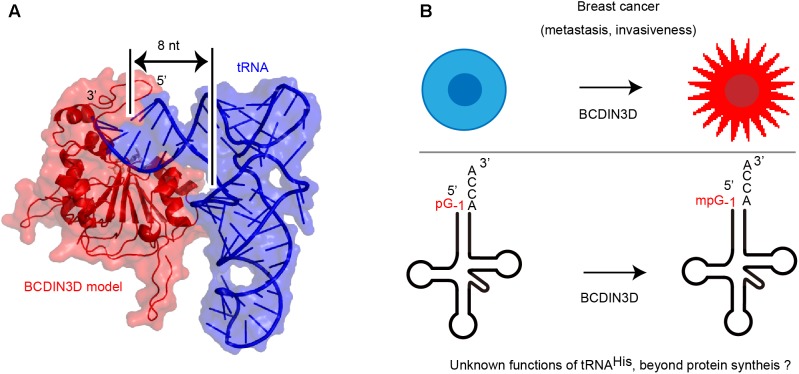
**(A)** A model of human Bicoid interacting 3 domain containing RNA methyltransferase (BCDIN3D)-tRNA^His^ complex structure. Human BCDIN3D structure was modeled using SWISS-MODEL (Biasini et al., 2014), based on the structure of the catalytic domain of methylphosphate capping enzyme (MePCE) (Jeronimo et al., 2007) as a template model. tRNA was manually docked onto the surface of the modeled BCDIN3D and the 5′-terminal of tRNA can enter the catalytic SAM binding site. BCDIN3D measures the length of the acceptor helix of tRNA. **(B)** A potential association between methylation of tRNA^His^ and breast cancer. tRNA^His^ might have unknown functions, beyond its established function in protein synthesis.

The structure of human BCDIN3D is still unclear. The amino acid sequence of BCDIN3D is homologous to that of the catalytic domain of methylphosphate capping enzyme (MePCE), which uses SAM to transfer a methyl group onto the γ-phosphate of the 5′-guanosine of 7SK RNA (Jeronimo et al., 2007; Shuman, 2007). Structural modeling of human BCDIN3D using the catalytic domain of MePCE and possible tRNA-binding modeling suggest that BCDIN3D recognizes the acceptor stem of tRNA^His^ and measures the length of acceptor helix of tRNA^His^ (**Figure 2A**). Only the 5′-end of tRNA with an 8-nucleotide-long acceptor helix and G_-1_:A_72_ mis-pairing at the top the acceptor helix could enter the catalytic pocket of BCDIN3D, and the 5′-phosphate would be monomethylated. The mechanism underlying the recognition of tRNA by BCDIN3D would differ from those for tRNA recognition by the CCA-adding enzymes (Tomita et al., 2004; Yamashita et al., 2014, 2015; Yamashita and Tomita, 2016),which recognize the TΨC loop of tRNA.

The kinetics of methylation of tRNA^His^ mutants and the structural model of BCDIN3D and tRNA^His^ complex also suggest that human BCDIN3D is tRNA^His^-specific 5′-monophosphate methyltransferase, and cytoplasmic tRNA^His^ is a primary target of human BCDIN3D.

## The Biological Role of 5′-Methylation of Cytoplasmic tRna^His^

The biological role of 5′-monomethylphosphate of cytoplasmic tRNA^His^ remains unclear. While the 5′-monomethylphosphate of cytoplasmic tRNA^His^ decreases the affinity of tRNA^His^ toward histidyl-tRNA synthetase (Martinez et al., 2017), as expected from the complex structure of bacterial histidyl-tRNA synthetase with tRNA^His^ (Tian et al., 2015) and biochemical evaluation (Fromant et al., 2000), the overall aminoacylation efficiency is not affected by the modification. The steady-state level of cytoplasmic tRNA^His^ in BCDIN3D-knockout cells and its parental HEK293T cells are not significantly different. Furthermore, the stabilities of cytoplasmic tRNA^His^ from HEK293T cells and BCDIN3D-knockout cells after treatment with actinomycin-D do not show significant differences (Martinez et al., 2017). However, 5′-monomethylmonophosphate protects cytoplasmic tRNA^His^ from degradation *in vitro* in cytoplasmic cell extracts. Thus, methylation of 5′-monophosphate of cytoplasmic tRNA^His^ might be involved in its stability under specific conditions or in certain biological processes.

## Perspective

The correlation between methylation of the 5′-monophosphate group of cytoplasmic tRNA^His^ and tumorigenic phenotype of breast cancer remains unknown (**Figure 2B**). tRNAs are involved in various biological process in cells (Sobala and Hutvagner, 2011; Raina and Ibba, 2014; Megel et al., 2015; Kumar et al., 2016; Park and Kim, 2018; Schimmel, 2018) beyond their established functions, as adaptors in protein synthesis.

In breast cancer cells, initiator tRNA^Met^ is reportedly upregulated (Pavon-Eternod et al., 2013). Furthermore, in highly metastatic breast cancer cells, the upregulation of specific tRNAs, such as tRNA^Glu^UUC and tRNA^Arg^CCG, stabilizes mRNAs containing the corresponding codons and enhances translation (Goodarzi et al., 2016). However, knockout of BCDIN3D in HEK293T does not affect the steady-state level of cytoplasmic tRNA^His^ (Martinez et al., 2017). Thus, 5′-monophosphate methylation of cytoplasmic tRNA^His^ would not enhance the translation of specific mRNAs, although this warrants further investigation. Small RNA fragments have reportedly been derived from tRNAs, i.e., tRNA fragments (tRFs), and participate in various cellular functions (Sobala and Hutvagner, 2011; Raina and Ibba, 2014; Kumar et al., 2016). Under various cellular stress conditions, tRFs are often produced (Ivanov et al., 2011, 2014; Durdevic et al., 2013; Durdevic and Schaefer, 2013; Gebetsberger and Polacek, 2013). In breast and prostate cancer, specific tRNAs, such as cytoplasmic tRNA^Lys^ and tRNA^His^, are cleaved by angiogenin, and the tRNA half fragments are abundantly expressed in a sex hormone-dependent manner (Honda et al., 2015). These tRNA half fragments also promote proliferation of breast and prostate cancer cells by a yet unknown mechanism. In human and mouse cells, 3′- or 5′- terminal tRFs (3′-tRF or 5′-tRF) are produced and accumulate in an asymmetric manner. These tRFs associate with Ago2 and the tRFs probably serve as typical miRNAs. The 3′-tRF, but not the 5′-tRF, derived from cytoplasmic tRNA^His^ is complementary to human endogenous retroviral sequences in the genome (Li et al., 2012).

It would be noteworthy to assume that 5′-monomethylation of 5′-phosphate of tRNA^His^ regulates the expression of the tRNA half fragments and/or tRFs derived from tRNA^His^ in breast cancer cells or under specific biological or stress conditions. The production of tRNA half fragments and/or tRFs, in turn, might regulate the genes involved in tumorigenesis in breast cancers. Future studies are required to understand whether methylation of the 5′-phosphate group of tRNA^His^ by BCDIN3D is involved in the tumorigenic phenotype of breast cancer and other cancers and to potentially elucidate the unknown functions of tRNAs, beyond their established functions.

## Author Contributions

All authors listed have made a substantial, direct and intellectual contribution to the work, and approved it for publication.

## Conflict of Interest Statement

The authors declare that the research was conducted in the absence of any commercial or financial relationships that could be construed as a potential conflict of interest.
